# Hemimasticatory spasm: a series of 17 cases and a comprehensive review of the literature

**DOI:** 10.3389/fneur.2024.1377289

**Published:** 2024-03-18

**Authors:** Kazuya Yoshida

**Affiliations:** Department of Oral and Maxillofacial Surgery, National Hospital Organization, Kyoto Medical Center, Kyoto, Japan

**Keywords:** hemimasticatory spasm, morphea, masseter muscle, facial hemiatrophy, scleroderma, Parry–Romberg syndrome, treatment, review

## Abstract

Hemimasticatory spasm (HMS) is a rare movement disorder characterized by paroxysmal spasms or twitches of the unilateral jaw-closing muscles. This study aimed to comprehensively evaluate the clinical features of patients with HMS. Data from 17 patients newly diagnosed with HMS (12 females and 5 males; mean age at onset: 46.7 years) who visited our department were retrospectively analyzed, and a literature search based on electronic medical databases from their inception until November 30, 2023, was conducted. A manual search was conducted for articles cited in the related literature. A total of 117 cases (72 females and 45 males; mean age at onset: 37.1 years) from 57 studies were analyzed. The muscles involved were the masseter (97.4%), temporalis (47.9%), and medial pterygoid (6%). Morphea or scleroderma was observed in 23.9% of the patients, and facial hemiatrophy in 27.4%. In 17.9% of the cases, Parry–Romberg syndrome was either complicated or suspected. Typical electromyographic findings included the absence of a silent period during spasms (23.9%) and irregular brief bursts of multiple motor unit potentials. Oral medicines, such as clonazepam or carbamazepine, alleviated the symptoms for some patients but were often unsatisfactory. Botulinum toxin therapy was effective in most cases. Recently, microvascular decompression surgery is increasingly being used, resulting in complete relief in some cases. In conclusion, highly effective modalities are currently available, and it is necessary to raise awareness of HMS to ensure that it can be diagnosed and treated accurately by both medical and dental professionals.

## 1 Introduction

Hemimasticatory spasm (HMS) is characterized by intermittent paroxysmal contractions of the unilateral jaw-closing muscles, resulting in brief twitches and/or prolonged spasms (1–56). Involuntary movements can cause muscle pain, tongue or buccal mucosa biting (9, 18, 21, 25, 34, 35, 55), and temporomandibular joint dislocation (6, 18, 54), resulting in masticatory disturbances or dysarthria. HMS is occasionally accompanied by hemifacial atrophy or localized scleroderma (1, 2, 7, 10, 13, 15, 17, 25, 43, 47, 51). Scleroderma is a chronic connective tissue disease characterized by skin lesions. Hüter (1) first described HMS in 1848, and it has been further reported over the past century (2–4, 57). The most common triggers precipitating the spasms are chewing, talking, and laughing. Brief twitches and painful spasms can last for a few seconds to minutes. The mechanism underlying HMS remains unclear, although vascular compression or focal demyelination of the trigeminal nerve has been considered a plausible etiology (8, 11–13). There are some options for the treatment of HMS, including oral medication, botulinum neurotoxin (BoNT) therapy (12, 13, 15, 17, 19, 21, 22, 24, 25, 27, 28, 34, 36, 38, 39, 42–45, 47, 49), and microvascular decompression of the trigeminal motor root (31–33, 39–41, 50). BoNT injection into the affected muscles is believed to be the most effective option.

HMS is a rare condition. It may be diagnosed as a hemifacial spasm or unilateral oromandibular dystonia by medical professionals such as neurologists, neurosurgeons, or otolaryngologists. Patients with mild or moderate HMS visit dentists or oral surgeons. However, few dental professionals are familiar with HMS. Therefore, almost all patients are diagnosed with bruxism or temporomandibular disorders. HMS is underdiagnosed and therefore under-recognized. Here, 17 new cases of HMS are presented, and all previous cases of HMS are thoroughly reviewed to elucidate the clinical signs and treatment outcomes of this entity.

## 2 Materials and methods

### 2.1 Case series

**Figure 1:**
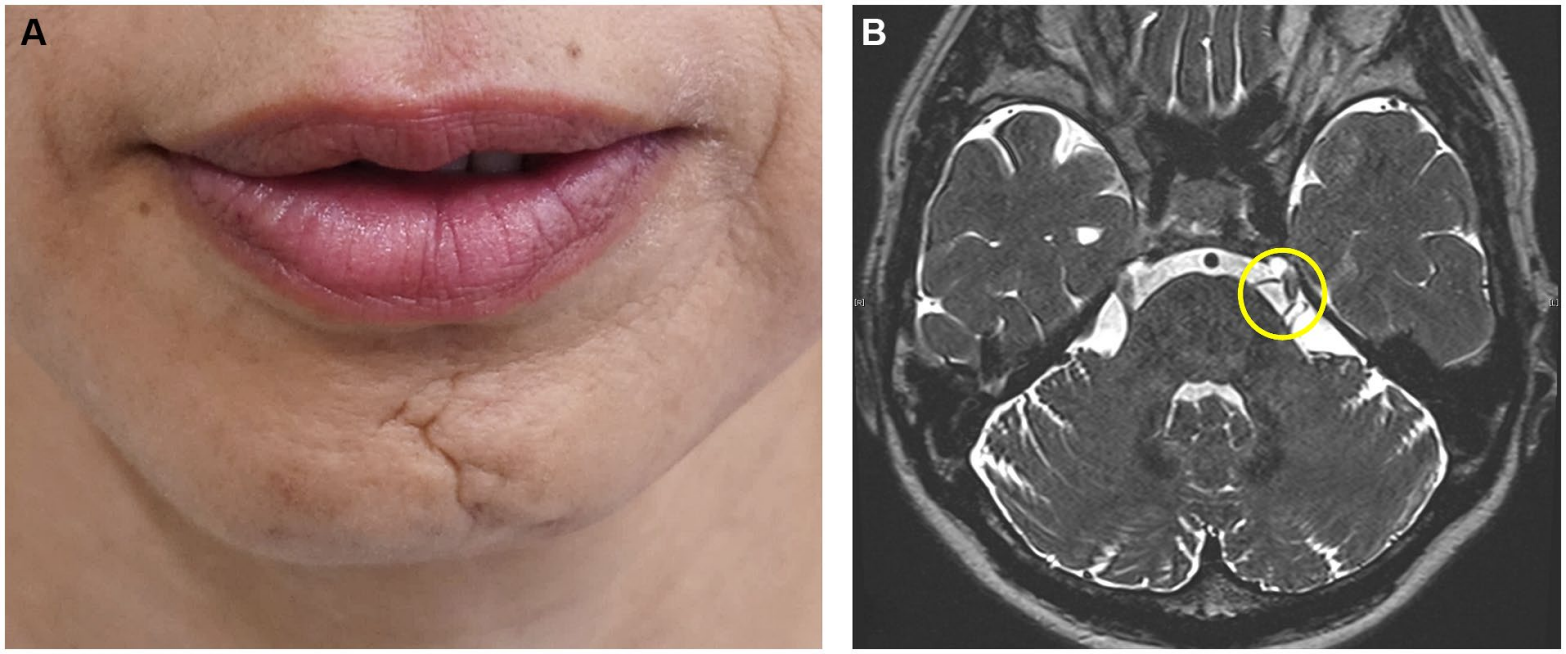
Characteristic clinical features of hemimasticatory spasm Linear scleroderma of the chin (**A**). Compression of the left trigeminal nerve by an artery is suspected on magnetic resonance imaging (**B**).

This study was conducted at a single institution and was based on the findings of a movement disorder specialist in the stomatognathic system. Among patients with complaints of involuntary movement, contracture, or pain in the masticatory, lingual, and/or lower facial muscles who visited our department between 2007 and 2022, patients with temporomandibular disorders, bruxism, multiple somatizations, hemifacial spasm, oromandibular dystonia, functional movement disorder, fibromyalgia, trigeminal neuralgia, or chronic pain were carefully examined and excluded from the analysis. The inclusion criteria were as follows: (i) paroxysmal, intermittent contractions of the jaw-closing muscles (Supplementary Video S1); (ii) brief twitches and/or prolonged spasms on one side; (iii) facial hemiatrophy or hypertrophy of the involved muscles; (iv) morphea or scleroderma (Figure 1A); (v) absence of a sensory trick, co-contraction, and morning benefit; and (vi) electromyography (EMG) findings. EMG was performed using surface or needle electrodes to examine the masseter, temporalis, and medial pterygoid muscles in some patients. Silent periods were examined by tapping with a reflex hammer in the mental region and by crushing raw macaroni (58). If a patient had more than four of these six features or findings, the patient was diagnosed with HMS.

Initially, oral medicines were tried unless they were proven to be ineffective by the referred physicians. Based on the response to oral medication, other treatments were conducted, such as muscle afferent block (MAB), occlusal splint, and BoNT therapy. MAB therapy was administered with the injection of a local anesthetic as described previously (59, 60). Three to five milliliters of 0.5% lidocaine (Xylocaine; Sandoz K.K., Tokyo, Japan) were injected into the masseter and/or temporalis muscle (59, 60). BoNT (onabotulinumtoxin A; Botox®; Allergan, Irvine, CA, United States; AbbVie, North Chicago, IL, USA) was administered to the muscles that cause spasms or involuntary contractions, as reported in previous studies (61, 62). Most patients noticed that their involuntary movements persisted during sleep, and occlusal splints were inserted, as reported previously (63). After an explanation of the possibility of vascular compression, if the patients desired, they were referred to neurosurgeons for further examination for indications of neurosurgical procedures (Figure 1B).

Patients received an explanation of the treatment plan and provided written informed consent. This study was performed in accordance with the tenets of the Declaration of Helsinki after obtaining approval from the Institutional Review Board and Ethics Committee of Kyoto Medical Center.

### 2.2 Review of literature

The literature search strategy was based on comprehensive electronic medical literature databases (PubMed, Embase, Google Scholar, and Japan Medical Abstracts Society) using the keywords (“hemimasticatory spasm” OR “hemifacial atrophy” OR “hemiatrophy” OR “muscle hypertrophy” OR “morphea” OR “scleroderma”) AND (“masticatory muscle” OR “masseter muscle” OR “jaw-closing muscle” OR “temporalis muscle”). Furthermore, a manual search was conducted for articles cited in related resources. Reports prior to November 30, 2023, identified in these databases or via a manual search with no language restrictions, were screened. Duplicate reports of the same patients were excluded. The exclusion criteria were records with missing fundamental information such as sex, age, or clinical presentation and those that were irrelevant to the purpose of the study. All reports were assessed for eligibility and reviewed by the author.

### 2.3 Analysis

Fundamental clinical data were evaluated and analyzed, including age at onset, sex, affected side, duration, involved muscles, muscle pain during spasm, suppression of spasm, existence of characteristic clinical features (morphea or scleroderma, facial hemiatrophy, and muscle hypertrophy), clinical features of involuntary movements, precipitating factors, results of electrophysiological study, EMG findings, possible etiology, treatments, oral medicines, BoNT therapy, injected muscles, units of BoNT, frequency of injections, surgical treatments, and follow-up (Supplementary Tables S1, S2).

### 2.4 Statistical analysis

Fisher’s exact test was performed to assess the statistical significance of differences in distribution. A two-tailed unpaired t-test was used to evaluate the changes. All analyses were performed using the SPSS statistical software package for Windows (version 24.0; SPSS Japan, Inc., Tokyo, Japan). The null hypothesis was rejected at a 5% significance level (*p* < 0.05).

## 3 Results

The demographic and clinical data of all 117 patients, including those of this review, are presented in Supplementary Table S1. The examinations and treatments are described in Supplementary Table S2.

### 3.1 Results of 17 novel cases

**Figure 2:**
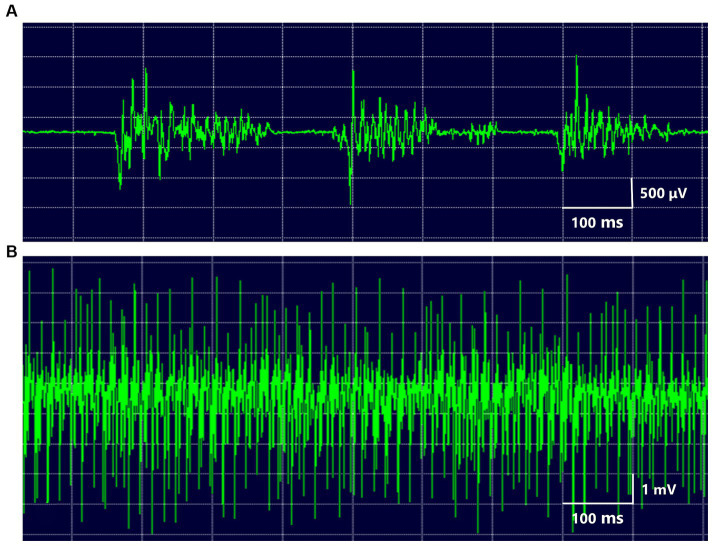
EMG of the masseter muscle several irregular brief bursts of multiple motor unit potentials were repeated in the masseter muscle **(A)**. During prolonged spasms, the masseter showed high-voltage motor potentials **(B)**.

The 17 patients (12 females and 5 males; mean age at onset ± standard deviation: 46.6 ± 16.1 years, Supplementary Table S1) complained of paroxysmal muscle contractions and pain on one side of the face (Supplementary Video S1). Two patients had facial morphea or scleroderma (Figure 1A). EMG of the masseter showed several irregular brief bursts of multiple motor unit potentials (Figure 2). In 15 patients (88.2%), the masseter reflex and silent period could not be elicited by tapping the chin with a reflex hammer during prolonged spasms with continuous bursts (Supplementary Table S2). Vascular compression was suspected in one patient (Figure 1B).

Prescribed oral medicines include clonazepam, baclofen, and tizanidine (Supplementary Table S2). MAB therapy was performed in six patients an average of 1.3 ± 0.52 times. BoNT was administered to 14 patients an average of 5.4 ± 4.5 times. Occlusal splints were inserted in nine patients. The mean subjective improvement rate was 72.4 ± 13.9%. The duration of follow-up was 49.6 ± 23.7 months.

### 3.2 Comprehensive review

#### 3.2.1 Demographic and clinical data

Our search yielded a total of 56 articles. The number of evaluated studies categorized by the original language was as follows: English, 46; Japanese, 2; French, 2; Russian, 2; German, 1; Spanish, 1; Chinese, 1; Korean, 1; and Latin, 1. The 56 articles including this report presented 117 patients (mean age at onset: 37.1 ± 13.9 years; range, 7–71 years) (Supplementary Table S1).

The demographic and clinical data of all cases, including those in this review, are summarized in Table 1. The mean duration of disease was 7.6 ± 7.5 years (range, 0.1–30 years). The patients comprised 72 women (61.5%) and 45 men (38.5%; Table 1). The 17 new patients (46.6 ± 16.1 years) were significantly (*p* = 0.012, unpaired t-test) older at disease onset than the other patients (37 ± 13.9 years). Among the patients, 60 (51.3%) were affected on the left side, 55 (47%) on the right side, and only 2 (1.7%) were bilaterally affected (45, 46). Precipitating factors included chewing in 35 patients (29.9%), speaking in 19 patients (16.2%), cold stimulus in 13 patients (11.1%), and jaw closing in 12 patients (10.3%). The muscles involved were the masseter in 56 patients (47.9%), the masseter + temporalis in 50 (42.7%), and the masseter + temporalis + medial pterygoid in 4 (3.4%) (Table 1). The masseter, temporalis, medial pterygoid, and tongue muscles were involved in 97.4, 47.9, 6, and 1.7% of patients, respectively. Forty-seven patients (40.2%) experienced muscle pain during the spasms (Supplementary Table S1). In 22 patients (18.8%), the spasms were suppressed by opening the mouth.

**Table 1:** Summary of demographic and clinical data of all 117 cases.

Age at onset (years), [mean ± SD, range]	37.1 ± 13, 7–71
Sex, [*n* (%)]	Female; *n* = 72 (61.5%), male; *n* = 45 (38.5%)
Affected side, [*n* (%)]	Left; *n* = 60 (51.3%), right; *n* = 55 (47%), bilateral; *n* = 2 (1.7%)
Duration (years), [mean ± SD, range]	7.6 ± 7.5, 0.1–30
Involved muscles, [*n* (%)]	Masseter; *n* = 56 (47.9%), masseter + temporalis; *n* = 50 (42.7%), masseter + temporalis + medial pterygoid; *n* = 4 (3.4%), temporalis; *n* = 2 (1.7%), masseter + temporalis + medial pterygoid; *n* = 2 (1.7%), masseter + temporalis + tongue muscle; *n* = 2 (1.7%), medial pterygoid; *n* = 1 (0.9%)
Precipitating factors, [*n* (%)]	Chewing; *n* = 35 (29.9%), speaking; *n* = 19 (16.2%), cold stimulus; *n* = 13 (11.1%), jaw closing; *n* = 12 (10.3%), none; *n* = 10 (8.5%), excitement; *n* = 5 (4.3%), laughing; *n* = 5 (4.3%), stress; *n* = 4 (3.4%), others; *n* = 2 (1.7%), NR; *n* = 22 (18.8%)
Muscle pain during spasm, [*n* (%)]	Yes; *n* = 47 (40.2%), no; *n* = 11 (9.4%), NR; *n* = 59 (50.4%)
How to suppress spasm, [*n* (%)]	Opening the mouth; *n* = 22 (18.8%), none; *n* = 4 (3.4%), NR; *n* = 91 (77.8%)
Other associated conditions, [*n* (%)]	Parry–Romberg syndrome; *n* = 21 (17.9%), pregnancy; *n* = 3 (2.6%), TMJ dislocation; *n* = 3 (2.6%), none; *n* = 11 (9.4%); NR; *n* = 42 (35.9%)
Medical history, [*n* (%)]	Dental infection; *n* = 9 (7.7%), gynecological diseases; *n* = 6 (5.1%), thyroid diseases; *n* = 3 (2.6%), lymphoma; *n* = 2 (1.7%), trauma; *n* = 2 (1.7%), none; *n* = 10 (8.5%), NR; *n* = 55 (47%)
Morphea or scleroderma, [*n* (%)]	Yes; *n* = 28 (23.9%), no; *n* = 37 (31.6%), NR; *n* = 52 (44.4%)
Facial hemiatrophy, [*n* (%)]	Yes; *n* = 32 (27.4%), no; *n* = 59 (50.4%), NR; *n* = 26 (22.2%)
Muscle hypertrophy, [*n* (%)]	Yes; *n* = 65 (55.6%) [masseter; *n* = 24 (20.5%), masseter + temporalis; *n* = 15 (12%), bilateral masseter; *n* = 2 (1.7%), temporalis; *n* = 2 (1.7%),], no; *n* = 38 (32.5%), NR; *n* = 14 (12%)
Possible etiology, [*n* (%)]	Vascular compression; *n* = 34 (29.1%), adhesion of nerve; *n* = 6 (5.1%), hormonal change; *n* = 3 (2.6%), demyelination by deep tissues; *n* = 3 (2.6%), idiopathic; *n* = 19 (16.2%), NR; *n* = 42 (35.9%)
Results of electrophysiological study, [*n* (%)]	Normal blink reflex; *n* = 31 (26.5%), absence of silent period; *n* = 27 (23.1%), absent or decreased jaw reflex; *n* = 18 (15.4%), normal jaw reflex; *n* = 8 (6.8%), NR; *n* = 39 (33.3%)

SD, standard deviation; NR, not reported; TMJ, temporomandibular joint.

Morphea or scleroderma was observed in 28 patients (23.9%) (Supplementary Table S1). In 10 cases, HMS developed a mean of 6.5 ± 5.1 years after the onset of morphea or scleroderma; in two cases, it developed simultaneously; and in one case, it developed 2 months before the onset of HMS. Facial hemiatrophy was observed in 32 patients (27.4%). Parry–Romberg syndrome (progressive hemifacial atrophy) was observed in 21 patients (17.9%). Of the 21 cases from 1848 to 2000, 16 (76.2%) had Parry–Romberg syndrome; of the 95 cases from 2001 to 2023, only five (5.3%) had Parry–Romberg syndrome, which was significantly (*p* < 0.0001, Fisher’s exact test) fewer. The patients with Parry–Romberg syndrome (27.5 ± 10.1 years) were significantly (*p* < 0.001, unpaired t-test) younger than the other patients (40 ± 14.8 years). In 10 patients, HMS developed a mean of 3.7 ± 3.4 years after the onset of Parry–Romberg syndrome; in five cases, it developed simultaneously; and in three cases, it developed a mean of 1.6 ± 1.2 years before the onset of HMS. In three cases, the symptoms worsened during pregnancy and were relieved after childbirth (21, 35, 47). The silent period was not elicited during spasms in 28 patients (23.9%).

#### 3.2.2 Results of treatments

A summary of the treatment results for all 117 patients is presented in Table 2. Oral medications were administered to 76 patients (65%). BoNT therapy was performed in 48 patients (41%), and surgical procedures were performed in 34 patients (29.1%) (Table 2; Supplementary Table S2). Splints were inserted in 12 patients (10.3%), and MAB therapy was attempted in 7 cases (6%). Oral medications included carbamazepine (21.4%), phenytoin (13.7%), clonazepam (10.3%), baclofen (6.8%), diazepam (3.4%), gabapentine (1.7%), and tolperisone (1.7%). BoNT therapy was administered into the masseter + temporalis (14.5%) and masseter (14.5%) a mean of 5.1 times. The injected muscles were the masseter (39.3%), temporalis (21.4%), and medial pterygoid (4.3%). Of the 48 patients treated with BoNT, 19 (17, 21, 24, 55) including 14 in this study received Botox; 3 (15, 22) received Dysport (Oculinum) (13), Myotox (50), and incobotulinumtoxin (42) in one case each. BoNT formulation was not described in 23 cases. The surgical procedures included microvascular decompression (17.9%), temporomandibular joint arthroscopic-assisted masseter nerve avulsion (6%), neurotomy (5.1%), and highly selective rhizotomy (2.6%). The duration of follow-up was 42.2 ± 44.7 months.

**Table 2:** Summary of results of treatments of all 117 cases.

Treatment, [*n* (%)]	Oral medication; *n* = 76 (65%) BoNT therapy; *n* = 48 (41%) Surgery; *n* = 34 (29.1%) Splint; *n* = 12 (10.3%) Muscle afferent block; *n* = 7 (6%)
Oral medicines, [*n* (%)]	Carbamazepine; *n* = 25 (21.4%), phenytoin; *n* = 16 (13.7%), clonazepam; *n* = 12 (10.3%), baclofen; *n* = 8 (6.8%), diazepam; *n* = 4 (3.4%), gabapentine; *n* = 2 (1.7%), tolperisone; *n* = 2 (1.7%)
BoNT therapy, [*n* (%)]	Masseter + temporalis; *n* = 17 (14.5%), masseter; *n* = 17 (14.5%), masseter + temporalis + others; *n* = 10 (8.5%), masseter + others; *n* = 2 (1.7%)
Injected muscles, [*n* (%)]	Masseter; *n* = 46 (39.3%), temporalis; *n* = 25 (21.4%), medial pterygoid; *n* = 5 (4.3%), posterior digastric; *n* = 5 (4.3%), others; *n* = 9 (7.7%)
Times of injections, [mean ± SD, range]	5.1 ± 4.7, 1–22
Surgical treatments, [*n* (%)]	Microvascular decompression; *n* = 22 (18.8%), partial resection of the trigeminal nerve motor branch; *n* = 7(6%), neurotomy; *n* = 6 (5.1%), TMJ arthroscopic-assisted masseter nerve avulsion; *n* = 4 (3.4%), highly selective rhizotomy; *n* = 3 (2.6%), others; *n* = 5 (4.3%)
Follow-up (months), [mean ± SD, range]	42.2 ± 44.7, 2–300

SD, standard deviation; BoNT, botulinum neurotoxin; TMJ, temporomandibular joint.

## 4 Discussion

This is the first comprehensive review of all previously published studies on HMS in all languages that were accessible through a database and manual search. This evaluation revealed the fundamental data, clinical features, and results of treatments for HMS.

Although the underlying mechanisms of HMS remain unclear, some possible etiologies include deep tissue changes caused by Parry–Romberg syndrome or scleroderma (13, 17), injury to or inflammation of the peripheral nerve (64), and pregnancy-related hormonal changes (21, 35, 47). Scleroderma is an autoimmune disease in which an overproduction of abnormal collagen results in normal tissues being replaced with scar tissue. Linear scleroderma *en coup de sabre* is a variant of localized scleroderma, which is characterized by atrophy and a furrow of the skin. Linear scleroderma can involve the lower face, Parry-Romberg syndrome, and upper face, with *en coup de sabre* so called owing to the resemblance to a “stroke from a sword” (65). Deep tissue changes resulting from linear scleroderma can cause localized injury to the motor fibers of the trigeminal nerve (13, 17). Parry–Romberg syndrome was first described by Parry (66) in 1825 and Romberg (67) in 1846. It is an infrequent craniofacial disorder characterized by progressive hemiatrophy of the skin, subcutaneous tissue, fat, and, in severe cases, the underlying muscle, cartilage, and bone (65, 68). Disease onset occurs within the first 20 years of life. Symptoms progress over a 2- to 10-year self-limiting period before spontaneous stabilization (64, 67). The pathogenesis of Parry–Romberg syndrome remains unknown; however, autoimmunity, trauma, and infection have been described. There is a close relationship between Parry–Romberg syndrome and linear scleroderma *en coup de sabre* (68). Progressive involution of the facial bones may cause hemifacial atrophy. A clear differentiation between Parry–Romberg syndrome and linear scleroderma *en coup de sabre* is often not possible, and the etiology of the two diseases may be similar (69). Deep tissue changes caused by Parry–Romberg syndrome or scleroderma might result in compression and focal demyelination of the motor branch of the trigeminal nerve (13, 17). Parry–Romberg syndrome was significantly more common among patients from 1848 to 2000 than from 2001 onwards. Additionally, the 17 new cases in this review were significantly older than those reported previously. These results suggest that early cases are often severe with marked hemifacial atrophy, and although mild cases of HMS have been reported, it takes time to make a definitive diagnosis. Injury to the peripheral nervous system has been reported to cause various movement disorders, such as dystonia, hemifacial spasm, tremor, myoclonus, tics, and parkinsonism (70–74). Although there is no confirmatory test to ascertain whether a movement disorder is genuinely induced by peripheral injury (74), several cases of HMS have been reported after severe dental inflammation (2, 4, 6). Recently, dental or oral surgical treatments have been reported to precede HMS (64). Three cases of HMS worsened during pregnancy and improved after childbirth (21, 35, 47). Thus, pregnancy-related hormonal changes can influence the mechanism responsible for HMS.

**Figure 3:**
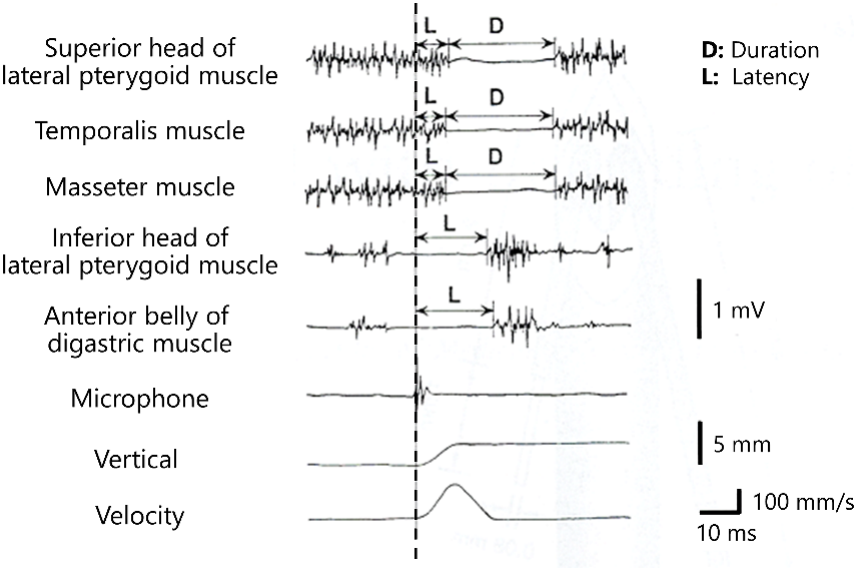
Silent periods and excitatory responses after unloading of the bite force Silent periods of the elevator muscles (the masseter muscle, temporalis muscles, and superior head of the lateral pterygoid muscle) and excitatory responses of the depressor muscles (inferior head of the lateral pterygoid muscle and anterior belly of the digastric muscle) are observed after crushing of food (dashed line).

The excitatory electrical activity of the trigeminal motor root or motor nucleus may play a major role in the development of HMS (8, 11–13). Kaufman first described the characteristic EMG findings of HMS (6). They are irregular bursts of motor unit potentials, correlating with twitches or spasms (Supplementary Table S1). Individual motor unit potentials have a high frequency of up to 200 Hz but are normal (6). The findings for HMS and hemifacial spasm are markedly similar. High-frequency motor unit discharges suggest a peripheral origin of the abnormal activity. Unlike unilateral oromandibular dystonia, HMS shows no co-contraction or overflow phenomenon. Electrophysiological findings revealed that blink reflexes were normal and masseter reflexes were absent or delayed in the examined patients (Supplementary Table S2). The results suggest the involvement of the Ia fibers, resulting in deafferentation of the muscle spindles and interruption of the ipsilateral monosynaptic masseter stretch arc (17). The most characteristic electrophysiological finding of HMS is the absence of silent periods during spasms. The silent period is a temporary period of cessation of the EMG activity of the jaw-closing muscles and appears with a latency of 7–10 ms after tooth contact during tapping movements, after hitting the chin during clenching, or after unloading of the bite force after food crushing (58). Silent periods of the jaw-closing muscles such as the masseter muscle and excitatory responses of the jaw-opening muscles such as the inferior head of the lateral pterygoid muscle are associated with unloading of the bite force during food crushing (Figure 3). A complete efferent block to the muscles is an exceptional and unique finding in HMS (13, 17). Impaired inhibition of masseter muscle contraction as a result of ectopic excitation may play a major role in the mechanisms leading to HMS. According to electrophysiological findings, HMS originates from either the motor root or the motor nucleus of the trigeminal nerve in a similar fashion to the postulated mechanism of involuntary activities in hemifacial spasm (11, 12). A trigeminal nerve branch to the masseter and temporalis muscles runs in a confined space between the lateral pterygoid muscle and the unyielding surface of the skull, where it might be easily compressed or stretched by deep tissue changes, resulting in focal demyelination of that branch (13). Hemifacial spasm is caused by vascular compression of the facial nerve near the nerve’s entry into the brain stem; conversely, HMS can be caused by compression or stretching of the distal mandibular nerve branch owing to deep tissue changes, such as facial hemiatrophy (13, 17). Neurological examination results and facial sensations were normal in most cases. The masseter, temporalis, medial pterygoid, and tongue muscles were involved in 97.4, 48.3, 6, and 1.7% of patients, respectively. Many researchers believe that the jaw-opening muscles are not affected. BoNT was administered to the other muscles (Supplementary Table S2). This was not for the spasm itself but for the compensatory activities of other muscles related to the abnormal activities of the jaw-closing muscles.

BoNT therapy for jaw-closing muscles is highly effective in improving spasms in patients with HMS. However, data regarding BoNT therapy for HMS are insufficient, and the author believes that the dose should be adjusted according to oromandibular dystonia (61). Repeated BoNT injections are likely to cause apparent facial hemiatrophy due to masseter muscle atrophy and masticatory disturbance owing to a markedly decreased bite force (56). During BoNT therapy on the jaw-closing muscles, the author always measures the occlusal force on the bilateral molars using an occlusal force meter (GM10, Nagano Keiki Co.; Tokyo, Japan) and is careful not to cause excessive reduction of the occlusal force (61). When the maximal occlusal force is less than 100 N, chewing is often difficult (75); therefore, the dose of BoNT should be reduced. Surprisingly, no attention has been paid to these aspects in previous reports. Most studies have shown only improvements in spasms and myalgia after several sessions of BoNT therapy with insufficient follow-up (Supplementary Table S1). In the future, attention should be focused not only on improving symptoms related to spasms but also on the long-term health-related quality of life of the patient, such as mastication and the esthetics of the facial appearance. As an alternative, MAB blocks muscle afferents for the treatment of focal dystonia, and a local injection of lidocaine reduces the effectiveness of muscle spindle afferents without causing unwanted weakness (59, 60). Previous studies have shown that MAB is significantly more effective than saline injections for oromandibular dystonia (59, 60). MAB therapy was used effectively in patients with oromandibular dystonia and masticatory muscle spasms (59, 60). Although further studies are necessary to clarify the mechanism underlying the effects of MAB therapy, local injections of lidocaine can block afferents in the muscle spindle (59, 60). The average number of muscle spindles on one side of the face is estimated at 342 for the temporal muscle, 114 for the masseter muscle, 59 for the medial pterygoid, and 6 for the lateral pterygoid muscle, although neither belly of the digastric muscle contains muscle spindles (76). In a previous study, the mean response of the jaw-closing muscles (70%) was significantly higher (*p* < 0.02) than that of the jaw-opening muscles (38%) (59). The significantly greater improvement in patients after injection into the jaw-closing muscles than in those after injection into the jaw-opening muscles might be related to the larger number of muscle spindles (59, 60). The jaw-closing muscles are rich in muscle spindles; therefore, MAB of the masseter muscle will be useful for patients with HMS. For patients who do not wish to undergo surgery and for whom expensive BoNT therapy is not feasible, a combination of MAB therapy and splinting, which improves muscle pain but does not cause muscle atrophy, has also been reported as an alternative (56).

Sensory trick splints have been reported to be effective in patients with oromandibular dystonia who exhibit sensory tricks, especially jaw-closing dystonia (63). However, no sensory tricks were observed in patients with HMS. Involuntary contractions of HMS persist during sleep. Some patients are awakened due to involuntary contractions and related pain during sleep. Therefore, it is useful to insert an occlusal splint in patients with HMS during sleep. Approximately one-fifth of the patients with HMS in this review observed that forcing the mouth to open during a spasm can suppress the spasm (Supplementary Table S1). Opening the mouth may stretch the jaw-closing muscles, thereby affecting signals from the muscle spindles. Some cases in which dental splints resulted in a slight to moderate relief of HMS symptoms have been reported (18, 26, 56). Increasing the occlusal vertical dimension with a splint may slightly stretch the jaw-closing muscles, making spasms less likely to occur. More detailed studies with a larger number of patients are required to test this hypothesis.

Surgical interventions, such as microvascular decompression, may be effective for definitively diagnosed patients who show vascular compression. Vascular compression was suspected after magnetic resonance imaging and computed tomography in one patient (Figure 1B). However, as the symptoms were relieved after other conservative treatments, the patient did not desire neurosurgical procedures. One case of failure after microvascular decompression (32) and two cases of worsening after improvement (41) have been reported. Complications were rare in the 42 patients in whom surgical treatment was performed. However, mild atrophy of the temporal muscle occurred gradually in two patients undergoing complete amputation of the trigeminal motor nerve (41). Surgical effects should be followed up and evaluated after a minimum of 2 years after surgery (41). In the future, it seems necessary to conduct long-term follow-ups in more cases. Conservative treatment options should be considered for patients with relatively mild symptoms. In cases where a clear cause has been identified, such as vascular compression of the trigeminal nerve motor roots, it is beneficial for patients to remove the cause using surgical procedures, such as microvascular decompression. However, microvascular decompression has been ineffective in a few cases (32, 41), and there have been reports of symptom recurrence after surgical treatment (6, 12), suggesting the need for the selection of indications and long-term follow-up. Although there are several treatments for HMS, the effects of each treatment cannot be compared because the evaluation of their effectiveness is inconsistent (Supplementary Table S2). A rating scale has been developed for oromandibular dystonia, making an objective evaluation possible (77, 78), and thus a similar rating scale for HMS appears necessary.

The author has been treating and researching involuntary movements in the stomatognathic region for over 35 years, and a large number of patients are referred from Japan and overseas. HMS is considered to be rare. To date, approximately 100 cases have been reported (Supplementary Table S1). Most patients with relatively mild HMS consult a dentist or oral surgeon. However, most dentists and oral surgeons are unfamiliar with HMS. Unlike other movement disorders, symptoms of HMS persist during sleep and are likely to be diagnosed as sleep bruxism or temporomandibular disorders. Furthermore, occlusal splints are effective in some cases of HMS, and treatment is continued by dentists and oral surgeons. Even if patients consult neurologists, unless they are movement disorder specialists, a diagnosis of hemifacial spasm or unilateral jaw closing dystonia may be made. Although the most severe cases of progressive hemifacial atrophy are attended to by a neurologist or plastic surgeon, the vast majority of patients with relatively mild symptoms visit dentists or oral surgeons. In this study, strict inclusion criteria were used, limiting the study to 17 patients who could be definitively diagnosed with HMS. Several times as many suspected cases have been reported. The prevalence of HMS may thus be much higher than previously estimated, and a definitive diagnosis is extremely rare. All new patients presented here visited dentists or oral surgeons before being referred to our clinic. All patients were diagnosed with unilateral bruxism. Because the definition of bruxism remains ambiguous, many patients with various movement disorders of the stomatognathic system have been diagnosed with bruxism-related temporomandibular disorders. The author proposes that the definition of bruxism should include exclusion criteria for movement disorders. Both dental and medical professionals should be interested in this entity and integrate this information into their clinical practice.

Differential diagnoses of HMS include local/mechanical disorders of the mandible or temporomandibular joint, tetanus, trismus, focal motor epilepsy, unilateral oromandibular dystonia, tonic spasms of multiple sclerosis, tetany, hemifacial spasm, and facial myokymia (9). Characteristic features, such as unilateral painful paroxysmal spasms, scleroderma or morphea, and facial hemiatrophy, absence of co-contraction and sensory trick, and persistent symptoms during sleep should be examined for a differential diagnosis. EMG may provide important information such as loss of the silent period and irregular bursts with high-frequency motor units. In particular, the loss of the silent period is a unique finding specific to HMS. If EMG is readily available, a clinical diagnosis can be made quickly.

In conclusion, the diagnosis of HMS may not be difficult based on prior knowledge of the characteristic signs. Highly effective treatment methods such as BoNT therapy and microvascular decompression are now being used, and it is necessary to raise awareness of the disease for both dental and medical professionals to ensure that HMS can be diagnosed immediately.

## Data availability statement

The original contributions presented in the study are included in the article/Supplementary materials, further inquiries can be directed to the corresponding author.

## Ethics statement

The studies involving human participants were reviewed and approved by the Institutional Review Board and Ethics Committee of Kyoto Medical Center. Written informed consent to participate in this study was provided by the patient/participants or patient/participants legal guardian/next of kin.

## Author contributions

KY: Conceptualization, Data curation, Formal analysis, Funding acquisition, Investigation, Methodology, Project administration, Resources, Software, Supervision, Validation, Visualization, Writing – original draft, Writing – review & editing.
