# Supplementary material for: Hemimasticatory spasm: a series of 17 cases and a comprehensive review of the literature
Source: Front Neurol. 2024 Mar 18;15:1377289. doi: 10.3389/fneur.2024.1377289 (PMC10986637; doi:10.3389/fneur.2024.1377289)
Supplement: Supplementary file 1 [file Table_1.DOCX]

Supplementary Material

Hemimasticatory Spasm: A Comprehensive Review of the Literature and Series of 17 Novel Cases

Kazuya Yoshida^1^

*** Correspondence:** Kazuya Yoshida: [yoshida.kazuya.ut@mail.hosp.go.jp](mailto:yoshida.kazuya.ut@mail.hosp.go.jp) or [omdystonia@gmail.com](mailto:omdystonia@gmail.com)

**Supplementary Table 1**. Demographic and clinical data of all 117 cases

| **Author/year of publication** | **Age at onset (years)** | **Sex** | **Affected side** | **Duration (years)** | **Involved muscles** | **Clinical features** | **Precipitating factors** | **Muscle pain during spasm** | **How to suppress spasm** | **Other associated conditions** | **Medical history** | **Morphea or scleroderma** | **Facial hemiatrophy** | **Muscle hypertrophy** |
| --- | --- | --- | --- | --- | --- | --- | --- | --- | --- | --- | --- | --- | --- | --- |
| Hüter (1848)^1^ | 7 | M | L | 23 | M, T | Irregularly spaced masticatory muscle spasms on the L side initially causing the teeth to temporarily close tightly but disappeared again after several consecutive contractions | NR | NR | NR | Hyperesthesia in the first and second branches of the trigeminal nerve, atrophy of the L tongue and uvula, alopecia on the L side of the head, Parry–Romberg syndrome? | NR | Yes? | Yes | No |
| Courtet (1876)^2^ | 18 | M | R | 4 | M, T, tongue muscle | Contracture of the medial side of the mandible, suprahyoid, and infrahyoid muscles and the tongue, facial deviation, painful fibrous spasms of the masticatory muscles | Cold stimulus, alcohol | Yes | NR | Atrophy of the R tongue and soft palate, loss of beard on the affected side, Parry–Romberg syndrome? | Convulsion, severe dental infection, and lead poisoning | Yes | Yes | No |
| Sachs (1890)^3^ | 18 | F | L | 1 | M, T, tongue muscle | A series of painful clonic and then tonic contractions of the L T and M lasting a few seconds | Excitement, exposure to cold | Yes | NR | Atrophy of the L half of the tongue and floor of the mouth, Parry–Romberg syndrome? | None | Yes? | Yes | No |
| Krueger (1916)^4^ | 31 | M | L | 3 | M | Clonic and tonic contraction of the L T resulting in tongue biting lasting 15 to 60 s | Cold stimulus to the L cheek | No | NR | Parry–Romberg syndrome? | Severe bleeding after extraction of the upper molar, trauma to the L side of the head | No | Yes | No |
| Vivado (1928)^5^ | 28 | M | L | 0.1 | M | Continuous severely painful spasm in the L M | NR | Yes | NR | Syphilis meningitis, Parry–Romberg syndrome? | Hemiatrophy of the L face, arm, and leg | Yes? | Yes | No |
| Kaufman (1980)^6^ | 25 | F | L | 2 | M | Forceful spasms in the L cheek accompanied by intermittent corneal injection, pupil dilation, and tearing of the L eye, lasting less than a second | Anxiety, chewing | NR | Widely opening the mouth | Parry–Romberg syndrome, dislocation of the L TMJ | Intense local swelling after extraction of a L upper molar | NR | Yes | No |
| Lapresle & Desi (1982)^7^ | 15 | F | R | 13 | M | Painful spasm of the R M lasting for 10 to 30 min | NR | Yes | NR | Parry–Romberg syndrome, a crossed atrophy involving the L side of the body and the R side of the face and neck | Chronic polyarthritis | *En coup de sabre* | Yes | No |
| Thompson & Caroll (1983)^8^ | 57 | F | L | 3 | M, T | Prolonged jaw closure lasting for up to 1 min, numerous episodes occurring daily | None | NR | Opening the mouth | NR | Paresthesia and numbness in the L maxilla | No | No | M, T |
| Thompson et al. (1986)^9^ | 31 | F | R | 7 | M | Spasms of the muscle of the R jaw lasting for a second to several minutes | Mouth closure, eating | NR | Opening the mouth | Parry–Romberg syndrome | NR | Yes | No | M |
| Parisi et al. (1987)^10^ | 38 | F | R | 1 | M | Painful spasm in the R M | NR | Yes | NR | Parry–Romberg syndrome, sclerotic and other plaques on the R parietal zone of the scalp, on the R zygoma, nasobuccal groove, and frontal eminence | Common exanthematous disease, frontomaxillary sinusitis, dysodontiasis, and uterine fibromyomatosis | *En coup de sabre* | Yes | No |
| Yoshii et al. (1989)^11^ | 44 | M | L | 0.7 | M | Paroxysmal muscle contractions of the L M muscle lasting 1 to 2 s | Mental tension, occlusion | No | NR | None | NR | No | No | M |
| Auger et al. (1992)^12^ | 20 | F | R | 5 | M, T | Frequent painful spasms of R M and T causing the jaw to lock for up to 1 min | None | Yes | NR | NR | NR | NR | No | M, T |
|  | 17 | F | R | 14 | Mpt | Intermittent episodes of involuntary jaw closure associated with R jaw pain occurring every 2 to 4 min lasting approximately 30 s | None | Yes | NR | NR | NR | NR | No | NR |
|  | 20 | F | R | 5 | M, T | Involuntary spasms of the R M and T often causing the teeth to chatter | NR | NR | NR | NR | NR | NR | No | T |
| Cruccu et al. (1994)^13^ | 18 | M | L | 2 | T | Small twitches alternating with severe and painful prolonged contractions, lasting up to several minutes | NR | Yes | NR | Parry–Romberg syndrome | NR | NR | Yes | T |
|  | 44 | F | R | 6 | M, T | Brief twitches and prolonged painful spasms, occurring many times a day, lasting up to a few minutes | Spontaneously or more often, triggered by chewing, speaking, or other voluntary movements of the mouth and jaw | Yes | NR | Parry–Romberg syndrome | NR | Yes | Yes | M, T |
| Kim et al. (1994)^14^ | 44 | M | R | 7 | M | Intermittent involuntary painful contractions in the R temporomandibular region | Cold stimulus | Yes | NR | Parry–Romberg syndrome? | NR | NR | Yes | No |
| Ebersbach et al. (1995)^15^ | 26 | M | L | 10 | M, T | Irregular contractions ranging from brief twitching to prolonged spasms | NR | No | Jaw opening | Parry–Romberg syndrome? | NR | *En coup de sabre* | Yes | M, T |
|  | 26 | F | R | 4 | M, T | Painful spasms of the R masticatory muscles occurring up to 30 times per day | At first, only sporadically during mastication, later provoked by speaking | Yes | NR | Parry–Romberg syndrome?, vitiligo of the R side of face, neck, and chest and on the R arm | NR | Scleroderma | Yes | M, T |
| Bilen et al. (1999)^16^ | 46 | F | R | 2 | M | Masticatory spasms | NR | NR | NR | Parry–Romberg syndrome | None | Yes | Yes | NR |
| Kim et al. (2000)^17^ | 34 | F | R | 3 | M | Involuntary painful spasms lasting for only a few seconds but sometimes for up to a minute | Chewing | Yes | NR | Parry–Romberg syndrome? | NR | Localized scleroderma | Yes | M |
| Esteban et al. (2002)^18^ | 47 | F | L | 14 | M | Recurrent episodes of involuntary jaw closure occurring several times a day lasting up to a few minutes | Talking, laughing, and chewing | Yes | Open position with the tip of a finger interposed between the teeth | Acute pain and stiffness over the L temporomandibular region, frequent tongue and oral mucosa biting, temporomandibular luxation | NR | No | No | M |
| Teive et al. (2002)^19^ | 44 | F | R | NR | M, T | Facial spasms and severe pain compromising the R temporal region and the R side of the face lasting 1 to 2 min | At first mastication; recently, spontaneously | Yes | NR | NR | Irregular menstrual cycles | No | NR | NR |
| Wang et al. (2004)^20^ | 38 | F | L | 1.5 | M | Involuntary clenching or twitching that lasts from a few seconds to several minutes, several to several dozen times a day | Tooth contact, cold, and psychological factors | NR | NR | NR | NR | No | No | No |
|  | 12 | M | R | 20 | M, T |  |  |  |  |  |  | Yes | No | M, T |
|  | 33 | M | R | 20 | M |  |  |  |  |  |  | No | No | M |
|  | 42 | F | L | 10 | M, T |  |  |  |  |  |  | No | No | No |
| Cersósimo et al. (2003)^21^ | 29 | F | R | 5 | M, T | Painful violent spasms of the R jaw-closing muscles causing abrupt closing of the jaw, often leading to biting of the tongue, lasting up to 5 min with more than 15 long-lasting spasms daily | Jaw movements | Yes | Opening the mouth | Eight months pregnant | NR | No | No | M, T |
| Mir et al. (2006)^22^ | 26 | M | L | 29 | M | Spasm of the L M lasting from seconds to 2 min | Physical stress and fatigue, initially in cold weather, later at any time | NR | NR | NR | NR | NR | No | No |
| Gunduz et al. (2007)^23^ | 62 | F | R | 0.1 | M, T | Involuntary spasms of the R jaw repeatedly leading to prolonged jaw closure | Chewing | NR | NR | Ischemic cerebro-vascular disease, pontin infarction, peripheric facial palsy of the R side | Hyperthyroidism | NR | No | No |
| Jiménez-Jiménez et al. (2008)^24^ | 40 | M | R | 5 | M, T | Sustained involuntary contractions of the R masticatory muscles | NR | NR | NR | NR | Bilateral hand dystonia with bilateral opercular lesions | NR | NR | NR |
| Kumar et al. (2008)^25^ | 49 | F | L | 5 | M, T | Painless, episodic L-sided closures, forceful enough to injure the tongue and cheek, lateral jaw deviation | Biting, chewing | No | NR | Biting of the tongue or buccal mucosa | Restless leg syndrome, hypothyroidism | Morphea | Yes | No |
| Arai (2009)^26^ | 30 | F | L | 10 | M | Painful intermittent spasms of the L jaw-closing muscles | Maximal voluntary clenching | Yes | NR | Local panatrophy | NR | No | No | No |
| Kim et al. (2009)^27^ | 42 | F | L | 0.5 | M | Paroxysmal spasms of the L M continued for about 10 s lasting for up to a few minutes | NR | Yes | NR | None | NR | No | No | M |
| Sinha et al. (2011)^28^ | 38 | M | R | 0.5 | M | Painful spasms occurring almost throughout the day, each episode lasting 5 to 15 s, at times lasting up to 30 s | Spontaneously and light tapping of the skin over the region | Yes | NR | NR | NR | No | No | M |
| Yaltho & Jankovic (2011)^29^ | 63 | F | L | 4 | M | Paroxysmal contractions of the L M | NR | NR | NR | NR | NR | NR | No | NR |
| Gopalakrishnan et al. (2011)^30^ | 56 | F | L | Six hours after surgery | M, T | Clonic involuntary L-sided jaw closure with deviation of the chin to the L lasting for a few seconds | Talking, chewing | NR | NR | L hearing loss, numbness involving L half of the face | Vestibular schwannoma | NR | No | No |
| Chon et al. (2012)^31^ | 40 | M | R | 14 | M, T | Pain and spasm of masticatory muscle on the R temporal and facial region several times a day, lasting 1 to 2 s | Chewing | Yes | NR | NR | NR | No | Yes | No |
| Wang et al. (2013)^32^ | 50 | F | L | From 4 months to 15 years | M | NR | NR | NR | NR | NR | NR | NR | NR | NR |
|  | 42 | F | R |  | M, T |  |  |  |  |  |  |  |  |  |
|  | 38 | M | R |  | M |  |  |  |  |  |  |  |  |  |
|  | 48 | F | R |  | M |  |  |  |  |  |  |  |  |  |
|  | 57 | F | L |  | M, T |  |  |  |  |  |  |  |  |  |
|  | 53 | F | R |  | M, T |  |  |  |  |  |  |  |  |  |
| Dou et al. (2014)^33^ | 45 | F | L | 11 | M | Involuntary painful spasms in the L M consisting of brief twitches to prolonged spasms | Chewing, excitement | Yes | NR | NR | NR | NR | Yes | No |
| Christie et al. (2014)^34^ | 32 | F | R | 30 | M | Strange sensations in the R preauricular region and involuntary contractions of the jaw with up to 30 episodes per day, causing sporadic biting injuries to the buccal mucosa and hampering jaw opening for as long as 20 s | Laughing, talking, or eating | NR | NR | None | NR | No | No | M, T |
| Panda et al. (2014)^35^ | 33 | F | L | 2 | M, T | Intermittent involuntary painful spasms and locking of the jaw for 2 to 3 min, resulting in frequent tongue bites | Eating, laughing, and normal conversation | Yes | NR | Parry–Romberg syndrome, pregnancy | NR | No | Yes | No |
| Pandey (2015)^36^ | 26 | M | R | 2 | M | Involuntary painful contractions of the R jaw, lasting for several seconds to minutes | Eating, talking | Yes | NR | NR | NR | NR | No | M |
| Yuan et al. (2015)^37^ | 45 | M | R | 9 | M, T | Irregular contractions of the R M and T, ranging from brief twitches to prolonged spasms occurring several times a day | Chewing | NR | Opening the mouth | NR | BoNT therapy and MVD at another hospital | NR | No | No |
|  | 50 | M | L | 10 | M | Sudden clenching of the teeth causing injuries to the tongue and oral mucosa, occurring many times a day | Chewing, speaking | Yes | NR | NR | NR | NR | NR | M |
| Kim et al. (2015)^38^ | 27 | F | R | NR | M | Sudden onset of spasms of the R M | NR | NR | NR | Parry–Romberg syndrome, temporomandibular joint pain, and noise | NR | Yes | Yes | No |
| Sun et al. (2016)^39^ | 15 | M | L | 7 | M | Intermittent spasms of the L M with 7 to 10 episodes a day lasting more than 10 min | Emotional stress, cold stimulus to the face | NR | NR | NR | NR | NR | Yes | No |
| Yan et al. (2017)^41^ | 38 | F | L | 2 | M, T | Paroxysmal L facial region with an attack frequency of more than 10 episodes per day, lasting 10 to 15 s | Chewing | Yes | NR | NR | NR | No | No | No |
| Wu et al. (2018)^40^ | 40 | M | L | 10 | M, T | Paroxysmal or involuntary transient convulsions or prolonged progressive spasms of single or multiple masticatory muscles unilaterally, ranging from a few seconds to a few minutes | Eating, chewing, talking laughing, and other motions | NR | NR | NR | NR | NR | No | No |
|  | 41 | F | R | 7 | T |  |  |  |  |  |  |  |  |  |
|  | 43 | F | L | 7 | M, T |  |  |  |  |  |  |  |  |  |
|  | 13 | F | L | 30 | M, T |  |  |  |  |  |  |  |  |  |
|  | 45 | M | R | 4 | M, T |  |  |  |  |  |  |  |  |  |
|  | 35 | F | R | 1.5 | M |  |  |  |  |  |  |  |  |  |
|  | 40 | F | L | 2 | M, T |  |  |  |  |  |  |  |  |  |
|  | 17 | F | L | 30 | M, T |  |  |  |  |  |  |  |  |  |
|  | 43 | F | L | 5 | M, T |  |  |  |  |  |  |  |  |  |
|  | 36 | F | R | 5 | M |  |  |  |  |  |  |  |  |  |
| Danisi & Guidi (2018)^42^ | 28 | M | L | 2 | M, T | Spasms resulting in teeth chattering and involuntary movements of the jaw up to several times an hour | At first randomly; after a few months, nearly constant | No | Tightening all the upper and lower facial musculature into a forceful grimace | NR | None | Linear scleroderma | No | M, T |
| Radhakrishnan et al (2019)^43^ | 21 | M | L | 4 | M, T | Brief or progressively prolonged painful spasms of single or multiple masticatory muscles of one side, lasting from a few seconds to several minutes | Activities such as chewing, talking, clenching of teeth, or voluntary tapping of the involved muscles | NR | NR | Lichen sclerosis et atrophicus with segmental morphea involving L side of the face and neck, back of trunk, and L upper and lower limbs | NR | Segmental morphea | Yes | No |
|  | 29 | M | L | 5 | M, T |  |  |  |  | NR |  | Localized scleroderma | Yes | No |
|  | 21 | M | L | 2 | M |  |  |  |  | NR |  | No | Yes | No |
|  | 33 | M | R | 6 | M, T |  |  |  |  | NR |  | No | No | M |
|  | 36 | F | R | 9 | M |  |  |  |  | NR |  | Localized scleroderma | No | M |
|  | 30 | F | R | 8 | M, T |  |  |  |  | NR |  | No | Yes | No |
|  | 29 | F | R | 5 | M |  |  |  |  | NR |  | No | No | M |
| Chen et al. (2020)^44^ | 27 | M | L | 7 | M | Severe tension and spasm of the L cheek | Biting, swallowing | NR | NR | Parry–Romberg syndrome | NR | No | Yes | No |
| Tavadyan et al. (2021)^45^ | 21 | F | R, L | 2 | M | Multiple painless spasms during the day, more often on the R sides, less frequently on the L, lasting 2 to 10 s, less often 30 to 40 s | NR | No | NR | NR | NR | NR | No | Bilateral M |
| Woo et al. (2021)^46^ | 55 | M | R, L | 0.5 | M, T | Brief twitches and slow irregular spasms of the M and T bilaterally, lasting between seconds and minutes | Chewing, clenching | Yes | NR | Hypereosinophilic syndrome | Self-injected placental extracts into the abdomen on more than 100 occasions | No | No | Bilateral M |
| Korenko et al. (2021) ^47^ | 10 | F | R | 28 | M | Excessive jaw closing on the R side accompanying destruction of tooth enamel | Chewing, speaking, and laughing | Yes | Opening the mouth | NR | NR | No | No | M |
| Ray et al. (2022)^48^ | 21 | M | L | 3 | M, T | Painless involuntary abnormal movements of the L cheek and lateral aspect of the head | NR | No | NR | Localized hyperpigmentation over the L cheek and face | NR | Morphea | NR | NR |
|  | 24 | F | R | 7 | M, T | Sudden painful clenching of R jaw and teeth at a rate of 1 to 2 times a day, lasting 10 to 20 s | Stress, cold climate, and fasting | Yes | NR | Pregnancy | NR | Morphea | Yes | No |
|  | 41 | M | R | 1 | M | Painless involuntary movements of the R half of the face | Opposition of both jaws, talking | No | NR | NR | Hodgkin's lymphoma | NR | No | M, T |
|  | 36 | F | L | 1.2 | M | Episodic painful spasms of jaw muscles (mainly L) | Talking, chewing | Yes | NR | NR | NR | Morphea | No | M |
| Zhang et al. (2022)^49^ | 40 | M | L | 2 | M | 1) Acute pain at rest and during functional activity; 2) paroxysmal or persistent and uncontrollable muscle contractions, leading to trismus; 3) accompanied by acute malocclusion or cheek-biting; and 4) no obvious space-occupying or inflammatory lesions in the maxillofacial examination | NR | Yes | NR | NR | NR | NR | NR | NR |
|  | 61 | F | R | 10 | M |  |  |  |  |  |  |  |  |  |
|  | 69 | M | L | 1 | M |  |  |  |  |  |  |  |  |  |
|  | 65 | M | R | 3 | M |  |  |  |  |  |  |  |  |  |
| Valieva et al. (2022)^50^ | 20 | F | L | 30 | M, T | Paroxysmal painless spasms of the L M and T lasting 10 to 20 s | Talking, spontaneously | No | NR | NR | NR | NR | No | M, T |
| Li et al. (2022)^51^ | 39 | F | R | 5 | M, T | NR | NR | NR | NR | NR | NR | NR | NR | NR |
|  | 25 | M | R | 20 | M |  | Weather changes |  |  |  | NR |  |  | NR |
|  | 30 | F | R | 10 | M, T |  | NR |  |  |  | NR |  |  | NR |
|  | 48 | F | L | 9 | M, Mpt |  | NR |  |  |  | NR |  |  | NR |
|  | 23 | M | R | 10 | M, T |  | NR |  |  |  | L nasal sinus cyst |  |  | NR |
|  | 41 | F | R | 10 | M, T |  | NR |  |  |  | Breast cancer |  |  | M, T |
| Mbodji et al. (2022)^52^ | 20 | F | R | 4 | M, T | Intermittent contractions of the T and M | Chewing | NR | NR | Parry–Romberg syndrome, frontal and brow capillary depilation, enophthalmia, lagophtalmia, and vitiligo lesions on the neck, the L scapula, axillary region, and forearm | NR | *En coup de sabre* | Yes | No |
| Xu et al. (2023)^53^ | 21 | M | L | 8 | M, T | Paroxysmal involuntary twitching of the L masticatory muscles accompanied by pain lasting for 3 to 5 s before spontaneously resolving | Cold stimulus, tension | Yes | NR | Parry–Romberg syndrome | NR | NR | Yes | M, T |
| Pillai et al. (2023)^54^ | 38 | M | L | 3 | M | NR | NR | NR | NR | NR | NR | NR | NR | NR |
|  | 51 | F | L | 11 | M |  |  |  |  |  |  |  |  |  |
|  | 68 | M | L | 2 | M |  |  |  |  |  |  |  |  |  |
|  | 62 | M | R | 4 | M |  |  |  |  |  |  |  |  |  |
|  | 49 | F | R | 11 | M |  |  |  |  |  |  |  |  |  |
|  | 65 | M | L | 3 | M |  |  |  |  |  |  |  |  |  |
| Koneru & Ondo (2023)^55^ | 30 | F | L | 7 | M, T | Severe pain and forceful L jaw clenching | NR | Yes | NR | TMJ dislocation, L frontal linear alopecia, atrophic skin changes at the L lateral mouth | Kidney tumor | Localized scleroderma | No | M, T |
| Yoshida (2024)^56^ | 49 | F | L | 2 | M | Paroxysmal involuntary twitching of the L M | Chewing, cold stimulus | Yes | Opening the mouth | Cramps | Ovarian cyst | Morphea | Yes | No |
| This study (2024) | 29 | M | L | 10 | M, Mpt | Paroxysmal involuntary clenching on the L side of the face | None | Yes | Opening the mouth | None | None | No | No | M |
|  | 26 | F | R | 8 | M | Rhythmic involuntary twitches of the R M | Clenching | Yes | Opening the mouth | None | None | No | No | M |
|  | 70 | M | L | 0.2 | M, T | Paroxysmal clenching of the L M and T | Chewing | Yes | Opening the mouth | None | Cerebral infarct, tooth extraction | No | No | No |
|  | 39 | F | L | 4.2 | M | Involuntary painful clenching of the L M | Speaking, chewing | Yes | None | None | None | No | No | No |
|  | 36 | F | L | 4 | M, T, Mpt | Paroxysmal painful contractions of the L M and T | None | Yes | Opening the mouth | Severe facial pain | None | No | No | M |
|  | 71 | F | L | 7 | M, T, Mpt | Involuntary painful contractions of the L side of the face | Chewing | Yes | None | L otalgia, tinnitus | Lymphoma, L tonsillectomy | No | No | M |
|  | 49 | F | R | 5.2 | M, Mpt | Paroxysmal painful clenching of the R M | Chewing | Yes | Opening the mouth | R side neck, shoulder, and back pain | Uterine fibroids, Hashimoto's disease | No | Yes | No |
|  | 50 | M | L | 1 | M | Paroxysmal rhythmic contractions of the L M | None | Yes | None | None | None | No | No | M |
|  | 33 | F | L | 10 | M, T | Painful chewing-like involuntary movements on the L side | Chewing | None | Opening the mouth | Discomfort in the L ear canal, involuntary movements of the pharynx | Endometriosis | Yes | No | M |
|  | 33 | M | L | 3 | M, T | Rhythmic involuntary jaw clenching of the L M and T | Tooth contact, chewing, speaking | Yes | Opening the mouth | None | None | No | No | M, T |
|  | 20 | F | L | 8 | M | Paroxysmal clenching of the L M | None | None | Opening the mouth | Extensive dental caries | Mixed connective tissue disease, complex regional pain syndrome type 1 | No | No | No |
|  | 64 | F | R | 0.8 | M | Paroxysmal contraction of the R M | Speaking, chewing | Yes | Opening the mouth | TMJ disorder | Dental treatment | No | No | No |
|  | 47 | M | R | 18 | M, T | Involuntary clenching of the R M and T | None | Yes | Opening the mouth | None | R mandibular condylar fracture | No | No | M |
|  | 48 | F | R | 0.7 | M, T | Paroxysmal clenching of the R M and T | Speaking, chewing | Yes | Opening the mouth | None | Uterine cancer | No | No | M |
|  | 62 | F | R | 8 | M, Mpt | Involuntary rhythmic clenching of the R M | Speaking, chewing | Yes | Opening the mouth | Trigeminal neuralgia | Trigeminal neuralgia surgery (MVD) | No | No | M |
|  | 48 | F | L | 3 | M | Paroxysmal rhythmic contraction of the L M | Jaw closing, cold stimulus | Yes | Opening the mouth | None | None | Yes | Yes | No |
|  | 68 | F | L | 10 | M | Paroxysmal clenching of the L M and T | None | Yes | None | Pain and discomfort in the lower L molar | Hypertension, cataract | No | No | M |

M, male; F, female; L, left; R, right; M, masseter, T, temporalis; Mpt, medial pterygoid; NR, not reported; BoNT, botulinum neurotoxin; MVD, microvascular decompression; TMJ, temporomandibular joint

**Supplementary Table 2**. Results of examination and treatments of all 117 cases

| **Author/year of publication** | **Age at onset (years)** | **Sex** | **Electrophysiological study** | **Electromygraphy findings** | **Possible etiology** | **Treatments** | **Oral medicines** | **BoNT therapy** | **Injected muscles** | **Units of BoNT** | | **Times of injections (times)** | | | **Surgical treatments** | | **Therapeutic effects** | | | | **Follow-up (months)** | |  |  |
| --- | --- | --- | --- | --- | --- | --- | --- | --- | --- | --- | --- | --- | --- | --- | --- | --- | --- | --- | --- | --- | --- | --- | --- | --- |
| Hüter (1848)^1^ | 7 | M | NR | NR | NR | NR | NR | NA | NA | NA | | NA | | | NA | | Lasted for 7 years with the same intensity and frequency, but then gradually became less frequent and milder | | | | NR | |  |  |
| Courtet (1876)^2^ | 18 | M | NR | NR | NR | Oral medication, electrical stimulation | NR | NA | NA | NA | | NA | | | NA | | No effect | | | | NR | |  |  |
| Sachs (1890)^3^ | 18 | F | NR | NR | NR | NR | NR | NA | NA | NA | | NA | | | NA | | NR | | | | NR | |  |  |
| Krueger (1916)^4^ | 31 | M | NR | NR | NR | NR | NR | NA | NA | NA | | NA | | | NA | | NR | | | | NR | |  |  |
| Vivado (1928)^5^ | 28 | M | NR | NR | Syphilis meningitis? | Mercury, bismas? | NA | NA | NA | NA | | NA | | | NA | | No spasm | | | | 3 | |  |  |
| Kaufman (1980)^6^ | 25 | F | Mild chronic denervation of the L M muscle | Spontaneous bursts of muscle activity lasting up to 100 ms occurring synchronously during the muscle twitches | Mandibular nerve injury? | Oral medication, surgery | Methocarbamol, diazepam, dantrolene sodium, phenytoin, and carbamazepine | NA | NA | NA | | NA | | | Rubbing the mandibular nerve with a nerve hook | | Oral medicines provided no benefit, postoperatively achieved complete relieve, later returned to the preoperative status | | | | 18 | |  |  |
| Lapresle & Desi (1982)^7^ | 15 | F | Normal motor and sensory conduction velocities and distal latencies | Anomalous, polyphasic motor unit potentials of variable amplitudes in the R M | NR | NR | NR | NA | NA | NA | | NA | | | NA | | NR | | | | NR | |  |  |
| Thompson & Caroll (1983)^8^ | 57 | F | Normal blink reflex, absence of jaw jerk reflex in the L M | Brief spontaneous spasms, comprising normal motor units discharging at high frequencies and a tendency to become repetitive, often in a crescendo fashion | Vascular compression? | Oral medication, cryosurgery | Diazepam, phenytoin, clonazepam, baclofen, valproate, haloperidol, and amitriptyline | NA | NA | NA | | NA | | | Cryosurgery of the motor root | | Oral medicines yielded no effect, temporary relief with surgery | | | | NR | |  |  |
| Thompson et al. (1986)^9^ | 31 | F | Normal blink reflex, prolonged latency of the M reflex on the R | Spontaneous activity in the R M during spasm, repetitive spontaneous bursts of motor unit discharges, varying in duration from 20 to 400 ms | NR | Oral medication, surgery | Carbamazepine, phenytoin | NA | NA | NA | | NA | | | M myotomy | | Significant reduction in severity of attacks with oral medicines, no improvement with surgery | | | | NR | |  |  |
| Parisi et al. (1987)^10^ | 38 | F | NR | Rapid discharges of high-frequency motor units | NR | Oral medication | Carbamazepine | NA | NA | NA | | NA | | | NA | | NR | | | | NR | |  |  |
| Yoshii et al. (1989)^11^ | 44 | M | Normal trigeminal nerve-evoked potential | Sudden onset and offset of bursts of spontaneous motor unit discharges in the L M and lateral and medial pterygoid muscles | NR | Oral medication | Clonazepam | NA | NA | NA | | NA | | | NA | | Slight relief | | | | NR | |  |  |
| Auger et al. (1992)^12^ | 20 | F | Normal blink and M reflexes, incomplete inhibition of M activity during spasms | Brief clonic discharges that merged into a continuous discharge during spasms, lasting from 10 to 200 ms | NR | Surgery, BoNT therapy | NR | Yes | M, T | NR | | NR | | | Sectioning trigeminal nerve rootlets | | Recurrence of involuntary spasms after substantial improvement | | | | 9 | |  |  |
|  | 17 | F | Normal blink and M reflexes, incomplete inhibition of M activity during spasms | Brief clonic discharges during spasms | NR | Oral medication | Carbamazepine | NA | NA | NA | | NA | | | NA | | Resolution of the symptoms | | | | NR | |  |  |
|  | 20 | F | Normal blink reflex | Brief clonic discharges during spasms in T | NR | Oral medication | Carbamazepine, phenytoin, and diazepam | NA | NA | NA | | NA | | | NA | | No effect with oral medicines | | | | 300 | |  |  |
| Cruccu et al. (1994)^13^ | 18 | M | Normal blink reflex, absence of jaw jerk reflex (silent periods) in the L T | Bursts of high-frequency multiple unit discharges (50 to 100 ms), recruitment of synchronized motor units leading to 60-Hz tonic activity lasting up to 2 min | Peripheral muscle nerve, focal demyelination by compression | Oral medication | Carbamazepine | NA | NA | NA | | NA | | | NA | | Moderate benefit | | | | NR | |  |  |
|  | 44 | F | Normal blink reflex, absence of jaw jerk reflex (silent periods) in the R M and T | Bursts of high-frequency multiple unit discharges (50 to 100 ms), recruitment of synchronized motor units leading to 70 Hz tonic activity lasting up to 30 s | Peripheral muscle nerve, focal demyelination by compression | Oral medication, injection of local anesthetics, BoNT therapy | Carbamazepine, diazepam | BoNT/A (Oculinum) | M, T | 30 to 50 | | 3 | | | NA | | No effect with oral medicines, clinical benefit with BoNT therapy | | | | NR | |  |  |
| Kim et al. (1994)^14^ | 44 | M | Normal blink and M inhibitory reflexes, incomplete silent period during spasms | Clonic discharge in the R M lasting 20 s during spasm | NR | Oral medication | Carbamazepine, phenytoin | NA | NA | NA | | NA | | | NA | | No improvement | | | | 3 | |  |  |
| Ebersbach et al. (1995)^15^ | 26 | M | Normal blink and M reflexes | Synchronous discharges of the L M and T with a duration of 50 to 500 ms, occurring at random intervals and correlating with brief visible twitches of the muscles | NR | BoNT therapy | No | BoNT/A (Dysport) | M, T | M, 40; T, 40 | | 2 | | | NA | | Comparable success | | | | NR | |  |  |
|  | 26 | F | Normal blink and M reflexes | Bursts occurring spontaneously or after provocation and lasting for 250 to 1,200 ms | NR | BoNT therapy | No | BoNT/A (Dysport) | M, T | M, 40; T, 100 | | 3 | | | NA | | Dramatic effect | | | | NR | |  |  |
| Bilen et al. (1999)^16^ | 46 | F | NR | NR | NR | Oral medication | Phenytoin | NA | NA | NA | | NA | | | NA | | Relief | | | | NR | |  |  |
| Kim et al. (2000)^17^ | 34 | F | Normal blink reflex, absence of M reflex and silent period in the R M | During spasms, brief bursts of motor unit potentials with normal shape discharging at high frequencies up to 200 Hz, lasting from 100 to 600 ms | Focal demyelination of motor branches of the trigeminal nerve owing to deep tissue changes | BoNT therapy | NR | BoNT/A (Botox) | M | 20 | | 2 | | | NA | | Almost complete disappearance of spasms | | | | NR | |  |  |
| Esteban et al. (2002)^18^ | 47 | F | Normal blink reflex, normal jaw reflexes in the first evaluation, delayed and reduced amplitude in the second, absence of silent period in the L M during spasm episodes | High voltage-high frequency discharges, both tonic or grouped, which usually followed a mild voluntary activity | An abnormal trigeminal hyperexcitability likely induced by the demyelinating lesion | Dental splint | NR | NA | NA | NA | | NA | | | NA | | Slight relief | | | | 168 | |  |  |
| Teive et al. (2002)^19^ | 44 | F | NR | Bursts of motor unit potentials, with a duration of 40 s and irregular frequency during spasms | NR | Oral medication, BoNT therapy | Carbamazepine, clonazepam, and gabapentin | BoNT/A (Botox) | M, T | 100 | | 2? | | | NA | | No effects with oral medicines, remarkable improvement with BoNT therapy | | | | NR | |  |  |
| Wang et al. (2004)^20^ | 38 | F | NR | Rhythmic discharges for 10 to 200 ms with a frequency of 3 to 5 times per second | NR | NR | NR | NR | NR | NR | | NR | | | NR | | NR | | | | NR | |  |  |
|  | 12 | M |  |  |  |  |  |  |  |  |  |  |  |  |  |  |  |  |  |  |  |  |  |  |
|  | 33 | M |  |  |  |  |  |  |  |  |  |  |  |  |  |  |  |  |  |  |  |  |  |  |
|  | 42 | F |  |  |  |  |  |  |  |  |  |  |  |  |  |  |  |  |  |  |  |  |  |  |
| Cersósimo et al. (2003)^21^ | 29 | F | NR | Brief and mild spasms (100 to 200 Hz) during involuntary activation in the M and T | Hormonal changes characteristic of pregnancy | BoNT therapy | No | BoNT/A (Botox) | M, T | M, 30; T, 60 | | 2 | | | NA | | Dramatic improvement with BoNT therapy, rapid and progressive relief after child birth | | | | 36 | |  |  |
| Mir et al. (2006)^22^ | 26 | M | NR | Paroxysmal spontaneous activity in the L M and T, brief bursts sometimes becoming more frequent, intense, and prolonged | Ectopic activity in the motor portion of the trigeminal nerve | Oral medication, BoNT therapy | Carbamazepine, dothiepin | BoNT/A (Dysport) | M | 80 | | NR | | | NA | | Slight effect with oral medicines, spasm-free with BoNT therapy | | | | NR | |  |  |
| Gunduz et al. (2007)^23^ | 62 | F | Absence of jaw jerk reflex, normal blink reflex | Irregular bursts of high-voltage motor unit potentials in T and M | Pontin infarction | BoNT therapy | NR | Yes | M, T | 70 | | 1 | | | NA | | Significant improvement | | | | NR | |  |  |
| Jiménez-Jiménez et al. (2008)^24^ | 40 | M | NR | Bursts of motor potential units at high frequencies in the R M and T during spasms | Biopercular infarct with Foix–Marie–Chavany syndrome | BoNT therapy | NR | BoNT/A (Botox) | M, T | M, 30; T, 30 | | 1 | | | NA | | Slight improvement | | | | Lost | |  |  |
| Kumar et al. (2008)^25^ | 49 | F | NR | High-frequency, irregular, motor unit potential bursts in association with jerking, involuntary movements | NR | Oral medication, BoNT therapy | Carbamazepine | Yes | M, T, Lpt | NR | | 1 | | | NA | | No effect with oral medicine, significant relief with BoNT therapy | | | | NR | |  |  |
| Arai (2009)^26^ | 30 | F | Normal blink reflex | Involuntary activity of the L M and T more than three times with maximal voluntary clenching during spasms | NR | Prosthetic treatment with removable partial denture that increased occlusal vertical dimension | Tolperisone | NA | NA | NA | | NA | | | NA | | Satisfactory relief: up to 3 spasms per month | | | | 120 | |  |  |
| Kim et al. (2009)^27^ | 42 | F | NR | Irregular bursts of motor unit potentials at 50 to 200 Hz during the period of spasm | NR | Oral medication, BoNT therapy | Phenytoin | BoNT | M | 20 to 50 | | Several times | | | NA | | Slight effect with oral medicine, substantial effect with BoNT therapy | | | | NR | |  |  |
| Sinha et al. (2011)^28^ | 38 | M | Prolonged latency of blink reflex, absence of masseteric silent period | NR | NR | BoNT therapy,　surgery | NR | BoNT | M | NR | | 1 | | | Debulking and stripping of the M muscle | | No recurrence | | | | 12 | |  |  |
| Yaltho & Jankovic (2011)^29^ | 63 | F | NR | NR | NR | NR | NR | NR | NR | NR | | NR | | | NR | | NR | | | | NR | |  |  |
| Gopalakrishnan et al. (2011)^30^ | 56 | F | NR | Irregular bursts of high-voltage motor potentials in the L T and M | Possibility of local nerve edema or focal demyelination | NA | NA | NA | NA | NA | | NA | | | NA | | Completely resolved by the sixth postoperative day, no motor dysfunction | | | | 3 | |  |  |
| Chon et al. (2012)^31^ | 40 | M | Absence of jaw jerk reflex, normal blink reflex | \|Irregular bursts of high-voltage motor unit potentials in the M and T during spasms | Compression of trigeminal motor root by trunk of superior cerebellar artery | Oral medication, BoNT therapy, and surgery | Carbamazepine, phenytoin | BoNT | M, T | NR | | 10 | | | MVD | | Improvement with BoNT therapy but recurred, improvement with surgery | | | | 20 | |  |  |
| Wang et al. (2013)^32^ | 50 | F | Normal corneal reflex, decreased mandibular reflex | NR | Superior cerebellar artery in 2 patients, anteroinferior cerebellar artery in 2 patients, and both in 2 patients | Surgery | NA | NA | NA | | NA | | NA | MVD | | | | | No relief | NR | | | | |
|  | 42 | F |  |  |  |  |  |  |  |  |  |  |  |  |  |  |  |  | Complete relief | Up for 4 to 58 months | | | | |
|  | 38 | M |  |  |  |  |  |  |  |  |  |  |  |  |  |  |  |  | Complete relief |  |  |  |  |  |
|  | 48 | F |  |  |  |  |  |  |  |  |  |  |  |  |  |  |  |  | Complete relief |  |  |  |  |  |
|  | 57 | F |  |  |  |  |  |  |  |  |  |  |  |  |  |  |  |  | Delayed relief |  |  |  |  |  |
|  | 53 | F |  |  |  |  |  |  |  |  |  |  |  |  |  |  |  |  | Complete relief |  |  |  |  |  |
| Dou et al. (2014)^33^ | 45 | F | NR | Brief bursts of motor unit potentials with normal shape discharging at high frequencies up to 200Hz during spasms | Compression by the superior cerebellar artery | Surgery | NR | NA | NA | NA | | NA | | | MVD | | Complete relief | | | | 7 | |  |  |
| Christie et al. (2014)^34^ | 32 | F | NR | Spontaneous activity consisting of repetitive, spontaneous bursts of motor unit discharges, ranging from 100 to 200 Hz | NR | BoNT therapy | NR | BoNT/A | M, T | M, 60; T, 40 | | Every 3-4 months | | | NA | | Excellent response | | | | NR | |  |  |
| Panda et al. (2014)^35^ | 33 | F | Absence of jaw jerk reflex, normal blink reflex | Spontaneous high-frequency, complex repetitive discharges with normal motor unit action even without spasm in the L M and T | NR | Oral medication | Carbamazepine | NA | NA | NA | | NA | | | NA | | Decreased spasm frequency from several times a day to once every week | | | | NR | |  |  |
| Pandey (2015)^36^ | 26 | M | NR | Variable duration bursts in the R M during spasms | NR | Oral medication, BoNT therapy | Antiepileptic or spasticity drugs | BoNT/A | M | 70 | | 1 | | | NA | | No effect with oral medicines, symptom-free with BoNT therapy | | | | 2 | |  |  |
| Yuan et al. (2015)^37^ | 45 | M | NR | NR | NR | Oral medication | Carbamazepine | NA | NA | NA | | NA | | | NA | | Excellent response | | | | 24 | |  |  |
|  | 50 | M | Normal blink reflex | Spasm of the L M without contraction of L T and R M | NR | Oral medication | Carbamazepine | NA | NA | NA | | NA | | | NA | | Good response | | | | 12 | |  |  |
| Kim et al. (2015)^38^ | 27 | F | NR | NR | NR | Oral medication, occlusal splint, arthrocentesis, BoNT therapy | Colchicine, eperisone | BoNT | M | NR | | NR | | | NA | | Spasms remained | | | | 36 | |  |  |
| Sun et al. (2016)^39^ | 15 | M | Prolonged latency of blink reflex, absence of masseteric silent period | Bursts of motor unit potentials that were synchronized with involuntary spasms | Nerve compression | Oral medication, acupuncture, BoNT therapy, and surgery | Carbamazepine, unknown oral medicines | BoNT | M, T | NR | | 8 | | | MVD | | No recurrence | | | | 12 | |  |  |
| Yan et al. (2017)^40^ | 38 | F | NR | Irregular high-voltage motor unit potentials during spasms | Trigeminal motor nerve rootlet compressed by the superior petrosal vein | Oral medication, surgery | Carbamazepine, other painkillers | NA | NA | NA | | NA | | | MVD | | No effect with oral medicine, no recurrence after surgery | | | | 6 | |  |  |
| Wu et al. (2018)^41^ | 40 | M | Normal corneal reflex, decreased mandibular reflex | High-frequency discharge and the disappearance of muscle rest during masticatory muscle spasm | Superior cerebellar artery | Acupuncture, oral medication, BoNT therapy without effects | Carbamazepine | NR | NR | NR | | NR | | | MVD, compete neurotomy | | No recurrence | | | | 60 | |  |  |
|  | 41 | F |  |  | Superior cerebellar artery |  |  |  |  |  |  |  |  |  | MVD | | Aggravated | | | | 60 | |  |  |
|  | 43 | F |  |  | Superior cerebellar artery |  |  |  |  |  |  |  |  |  | MVD | | Improved | | | | 60 | |  |  |
|  | 13 | F |  |  | Superior cerebellar artery, petrosal vein |  |  |  |  |  |  |  |  |  | MVD, selective partial neurotomy | | Recurred, improved | | | | 60 | |  |  |
|  | 45 | M |  |  | Petrosal vein |  |  |  |  |  |  |  |  |  | Neurotomy | | No recurrence | | | | 60 | |  |  |
|  | 35 | F |  |  | Superior cerebellar artery |  |  |  |  |  |  |  |  |  | MVD | | Disappeared | | | | 24 | |  |  |
|  | 40 | F |  |  | Superior cerebellar artery |  |  |  |  |  |  |  |  |  | MVD | | Worsened | | | | 24 | |  |  |
|  | 17 | F |  |  | Idiopathic |  |  |  |  |  |  |  |  |  | Neurotomy | | Disappeared | | | | 24 | |  |  |
|  | 43 | F |  |  | Superior cerebellar artery |  |  |  |  |  |  |  |  |  | MVD, selective partial neurotomy | | Improved | | | | 3 | |  |  |
|  | 36 | F |  |  | Petrosal vein |  |  |  |  |  |  |  |  |  | MVD, selective partial neurotomy | | Disappeared | | | | 3 | |  |  |
| Danisi & Guidi (2018)^42^ | 28 | M | NR | Grouped repetitive and sustained tonic discharges | Localized injury to the motor fibers from the deep tissue changes caused by linear scleroderma | BoNT therapy | NR | Incobotulinumtoxin A | M, T, frontalis, zygomatic major and minor, depressor anguli oris, depressor labii inferioris, risorius | 30 to 45 | | 22 | | | NA | | Marked relief | | | | NR | |  |  |
| Radhakrishnan et al (2019)^43^ | 21 | M | Normal blink reflex, loss of silent period during spasm | Spontaneous irregular bursts of high-frequency motor unit potentials | NR | Oral medication, BoNT therapy | Phenytoin | BoNT/A | M, T | M, 30; T, 15 | | 11 | | | NA | | At least 70% to 80% improvement of pain and spasms | | | | 36 | |  |  |
|  | 29 | M | Normal blink reflex |  |  |  |  |  | M, T | M, 20; T, 20 | | 3 | | |  |  |  |  |  |  | 18 | |  |  |
|  | 21 | M | Normal blink reflex |  |  |  |  |  | M | M, 20 | | 3 | | |  |  |  |  |  |  | 12 | |  |  |
|  | 33 | M | Normal blink reflex |  |  |  |  |  | M, T | M, 40; T, 20 | | 9 | | |  |  |  |  |  |  | 40 | |  |  |
|  | 36 | F | Normal blink reflex |  |  |  |  |  | M | M, 30 | | 8 | | |  |  |  |  |  |  | 30 | |  |  |
|  | 30 | F | Normal blink reflex, loss of silent period during spasms |  |  |  |  |  | M, T | M, 30; T, 20 | | 8 | | |  |  |  |  |  |  | 30 | |  |  |
|  | 29 | F | Normal blink reflex |  |  |  |  |  | M | M, 30 | | 7 | | |  |  |  |  |  |  | 28 | |  |  |
| Chen et al. (2020)^44^ | 27 | M | Normal blink reflex | Bursts of motor unit potentials synchronized with spasms | NR | Oral medication, BoNT therapy | Phenytoin | BoNT/A | M | 25 | | 2 | | | NA | | Significant relief | | | | NR | |  |  |
| Tavadyan et al. (2021)^45^ | 21 | F | Normal jaw reflex | Spontaneous burst of motor unit potentials that correlated with muscle spasms | NR | BoNT therapy | NR | BoNT/A | M | R M, 30, L M, 20 | | 2 | | | NA | | Excellent results | | | | NR | |  |  |
| Woo et al. (2021)^46^ | 55 | M | Normal blink reflex | Bursts of multiple motor unit potentials and asynchrony between bilateral muscle activities | NR | Oral medication | Carbamazepine | NA | NA | NA | | NA | | | NA | | Partial improvement | | | | NR | |  |  |
| Korenko et al. (2021) ^47^ | 10 | F | Normal blink reflex | Spontaneous burst of the R M | NR | BoNT therapy | Clonazepam | BoNT/A | Bilateral M | 50 | | 3 | | | NA | | Complete relief of pain | | | | NR | |  |  |
| Ray et al. (2022)^48^ | 21 | M | NR | NR | NR | Oral medication, BoNT therapy | Clonazepam | Yes | M | 100 | | Regular injection for 7 years | | | NA | | Spasms with a frequency of 1 to 2 per month | | | | 108 | |  |  |
|  | 24 | F | NR | NR | NR | Oral medication | Carbamazepine | NA | NA | NA | | NA | | | NA | | Asymptomatic | | | | 24 | |  |  |
|  | 41 | M | NR | NR | Superior cerebellar artery compressing R trigeminal nerve | Oral medication, BoNT therapy | Clonazepam, carbamazepine, and baclofen | Yes | NR | NR | | NR | | | NA | | 30% improvement | | | | 24 | |  |  |
|  | 36 | F | NR | NR | Vascular loop around R trigeminal nerve | Oral medication, BoNT therapy | Carbamazepine, clonazepam, and gabapentin | Yes | M | L, 30 to 35; R, 15 | | NR | | | NA | | Significant improvement | | | | 60 | |  |  |
| Zhang et al. (2022)^49^ | 40 | M | Increased recruitment phase potential and increased potential myoelectric activity with high-frequency discharge potential in the motor units that are synchronous with spasms, which is higher than the resting potential | High-frequency discharge potential in the recruitment phase | M nerve | Surgery | NR | NR | NR | NR | | NR | | | TMJ arthroscopic-assisted M nerve avulsion | | Complete remission | | | | 36 | |  |  |
|  | 61 | F |  |  |  |  |  |  |  |  |  |  |  |  |  |  |  |  |  |  |  |  |  |  |
|  | 69 | M |  |  |  |  |  |  |  |  |  |  |  |  |  |  |  |  |  |  |  |  |  |  |
|  | 65 | M |  |  |  |  |  |  |  |  |  |  |  |  |  |  |  |  |  |  |  |  |  |  |
| Valieva et al. (2022)^50^ | 20 | F | NR | Irregular bursts of motor unit potentials in the R M and T | NR | Oral medication, BoNT therapy | Tolperisone, carbamazepine | BoNT/A (Myotox) | Bilateral M, T, Mpt, and Lpt | R M, 30; L M, 40; R T, 20; L T, 40; R Mpt, 10; L Mpt, 10; R Lpt, 10; L Lpt, 10 | | NR | | | NA | | Improvement of symptoms | | | | NR | |  |  |
| Li et al. (2022)^51^ | 39 | F | NR | NR | Superior cerebellar artery | Oral medication, BoNT therapy, and surgery | Carbamazepine, clonazepam, and baclofen | BoNT/A was injected, but facial nerve paralysis occasionally occurred | | | | | | | | MVD | | No recurrence | | | | 60 | |  |
|  | 25 | M |  |  | Superior cerebellar artery, superior petrosal vein |  |  |  |  |  |  |  |  |  |  | MVD | | Obvious relief | | | | 60 | |  |
|  | 30 | F |  |  | Superior cerebellar artery attachment |  |  |  |  |  |  |  |  |  |  | MVD + trigeminal neurolysis + highly selective rhizotomy | | Partial relief | | | | 60 | |  |
|  | 48 | F |  |  | Superior cerebellar artery |  |  |  |  |  |  |  |  |  |  | MVD | | No recurrence | | | | 60 | |  |
|  | 23 | M |  |  | Idiopathic |  |  |  |  |  |  |  |  |  |  | Highly selective trigeminal rhizotomy (three branches) | | Obvious relief | | | | 36 | |  |
|  | 41 | F |  |  | Idiopathic |  |  |  |  |  |  |  |  |  |  | Highly selective trigeminal rhizotomy | | Disappearance of symptoms | | | | 12 | |  |
| Mbodji et al. (2022)^52^ | 20 | F | Normal blink reflex | Spontaneous, intermittent motor unit potentials | NR | Oral medication, BoNT therapy | Corticosteroids | Yes | NR | NR | | NR | | | NA | | Disappearance of spasms | | | | NR | |  |  |
| Xu et al. (2023)^53^ | 21 | M | NR | Synchronous bursts of motor unit activity in the L masticatory muscles during spasms | Demyelination changes from the effect of the facial involvement of Parry–Romberg syndrome on the trigeminal nerve motor branch | Surgery | NR | NR | NR | NR | | NR | | | Partial resection of the trigeminal nerve motor branch | | 50% improvement | | | | 24 | |  |  |
| Pillai et al. (2023)^54^ | 38 | M | NR | Greater frequency potential on the affected side than on the normal side | Adhesion of surrounding tissue to the M nerve? | Surgery | NR | NR | NR | NR | | NR | | | Partial resection of the trigeminal nerve motor branch | | Total remission | | | | 14 | |  |  |
|  | 51 | F |  |  |  |  |  |  |  |  |  |  |  |  |  |  |  |  |  |  |  |  |  |  |
|  | 68 | M |  |  |  |  |  |  |  |  |  |  |  |  |  |  |  |  |  |  |  |  |  |  |
|  | 62 | M |  |  |  |  |  |  |  |  |  |  |  |  |  |  |  |  |  |  |  |  |  |  |
|  | 49 | F |  |  |  |  |  |  |  |  |  |  |  |  |  |  |  |  |  |  |  |  |  |  |
|  | 65 | M |  |  |  |  |  |  |  |  |  |  |  |  |  |  |  |  |  |  |  |  |  |  |
| Koneru & Ondo (2023)^55^ | 30 | F | NR | NR | NR | BoNT therapy | NR | BoNT/A (Botox) | M, T | 200 | | Several times | | | NA | | Excellent improvement | | | | NR | |  |  |
| Yoshida (2023)^56^ | 49 | F | Loss of silent period during spasms | Several irregular brief bursts of multiple motor unit potentials in the L M | NR | Oral medication, occlusal splint, and MAB | Clonazepam | NA | NA | NA | | NA | | | NA | | 75% subjective improvement | | | | 12 | |  |  |
| This study (2024) | 29 | M | Loss of silent period during spasms | Irregular bursts of high-voltage motor unit potentials in the L M | Idiopathic | MAB, BoNT therapy | None | BoNT/A (Botox) | M, Mpt, Dp, Lpt, Da | M, 25; Mpt, 10; Dp, 5; Lpt, 10; Da, 5 | | 16 | | | NA | | 80% subjective improvement | | | | 50 | |  |  |
|  | 26 | F | Loss of silent period during spasms | Brief bursts of motor unit potentials in the R M synchronized with spasms | Idiopathic | BoNT therapy | None | BoNT/A (Botox) | M | M, 50 | | 8 | | | NA | | 90% subjective improvement | | | | 60 | |  |  |
|  | 70 | M | Loss of silent period during spasm | Irregular bursts of motor unit potentials in the R M and T | Idiopathic | Oral medication, occlusal splint, BoNT therapy | Baclofen | BoNT/A (Botox) | Bilateral M | M, 25 | | 1 | | | NA | | 70% subjective improvement | | | | 91 | |  |  |
|  | 39 | F | Loss of silent period during spasms | Irregular bursts of motor unit potentials in the L M | Idiopathic | Oral medication, occlusal splint | Baclofen | NA | NA | NA | | NA | | | NA | | 50% subjective improvement | | | | 98 | |  |  |
|  | 36 | F | Loss of silent period during spasm | Irregular bursts of high-voltage motor unit potentials in the L M and T | Idiopathic | Occlusal splint, BoNT therapy | None | BoNT/A (Botox) | M, T, Zygomatic major, Lpt, Mpt, Dp | M, 25; Zygomatic major, 2.5; Lpt, 15; Mpt, 10; Dp, 5 | | 9 | | | NA | | 80% subjective improvement | | | | 62 | |  |  |
|  | 71 | F | Loss of silent period during spasms | Irregular bursts of high-voltage motor unit potentials in the R M and T | Idiopathic | Oral medication, MAB, BoNT therapy | Tizanidine, baclofen | BoNT/A (Botox) | L M, T, Mpt, Dp, R M, | M, 25; T, 20; Mpt, 15; Dp, 5 | | 9 | | | NA | | 80% subjective improvement | | | | 46 | |  |  |
|  | 49 | F | Loss of silent period during spasms | Irregular bursts of high-voltage motor unit potentials in the R M | Idiopathic | MAB, BoNT therapy | None | BoNT/A (Botox) | M, Dp, Lpt, Mpt | M, 25; Dp, 5; Lpt, 20; Mpt, 10 | | 7 | | | NA | | 50% subjective improvement | | | | 50 | |  |  |
|  | 50 | M | Incomplete silent period during spasms | Irregular bursts of motor unit potentials in the L M synchronized with spasms | Idiopathic | Occlusal splint, BoNT therapy | None | BoNT/A (Botox) | M | M, 50 | | 2 | | | NA | | 90% subjective improvement | | | | 72 | |  |  |
|  | 33 | F | Loss of silent period during spasms | Brief irregular bursts of motor unit potentials in the L M and T synchronized with spasms | Idiopathic | Oral medication, occlusal splint, BoNT therapy | Baclofen, clonazepam | BoNT/A (Botox) | M, T, Lpt | M, 25; T, 20; Lpt, 15 | | 1 | | | NA | | 50% subjective improvement | | | | 76 | |  |  |
|  | 33 | M | Incomplete silent period during spasms | Brief bursts of motor unit potentials in the L M and T synchronized with spasms | Idiopathic | BoNT therapy | None | BoNT/A (Botox) | Bilateral M, T | M, 25; T, 20 | | 10 | | | NA | | 80% subjective improvement | | | | 36 | |  |  |
|  | 20 | F | Loss of silent period during spasms | Irregular bursts of motor unit potentials in the L M | Idiopathic | BoNT therapy | None | NA | NA | NA | | NA | | | NA | | 60% subjective improvement | | | | 36 | |  |  |
|  | 64 | F | Loss of silent period during spasms | Irregular bursts of high-voltage motor unit potentials in the R M | Idiopathic | Oral medication, MAB, BoNT therapy | Baclofen | BoNT/A (Botox) | M, Scm, Dp | M, 25; Scm, 15; Dp, 5 | | 1 | | | NA | | 70% subjective improvement | | | | 34 | |  |  |
|  | 47 | M | Loss of silent period during spasms | Irregular bursts of high-voltage motor unit potentials in the R M and T | Idiopathic | BoNT therapy | None | BoNT/A (Botox) | Bilateral M, R T | M, 25; T, 20 | | 3 | | | NA | | 80% subjective improvement | | | | 29 | |  |  |
|  | 48 | F | Loss of silent period during spasms | Irregular bursts of motor unit potentials in the R M and T | Idiopathic | Occlusal splint, BoNT therapy | None | BoNT/A (Botox) | Bilateral M, T, R mentalis | M, 25; T, 20; mentalis, 5 | | 3 | | | NA | | 90% subjective improvement | | | | 24 | |  |  |
|  | 62 | F | Loss of silent period during spasms | Irregular bursts of high-voltage motor unit potentials in the R M | Idiopathic | MAB, occlusal splint, BoNT therapy | None | BoNT/A (Botox) | R Lpt, M, bilateral T | M, 25; Lpt, 20; T, 20 | | 3 | | | NA | | 80% subjective improvement | | | | 24 | |  |  |
|  | 48 | F | Loss of silent period during spasms | Irregular brief bursts of motor unit potentials in the L M synchronized with spasms | Vascular compression | Oral medication, occlusal splint, MAB | Clonazepam | NA | NA | NA | | NA | | | NA | | 60% subjective improvement | | | | 23 | |  |  |
|  | 68 | F | Loss of silent period during spasms | Irregular bursts of high-voltage motor unit potentials in the L M | Idiopathic | Occlusal splint, BoNT therapy | None | BoNT/A (Botox) | M | M, 50 | | 3 | | | NA | | 70% subjective improvement | | | | 20 | |  |  |

M, male; F, female; L, left; R, right; M, masseter, T, temporalis; Mpt, medial pterygoid; Lpt, lateral pterygoid; Da, anterior digastric; Dp, posterior digastric; Scm, sternocleidomastoid; NR, not reported; NA, not applicable, BoNT, botulinum neurotoxin; MVD, microvascular decompression; MAB, muscle afferent block

**Video 1**. Muscle spasm of the right masseter muscle

Painful rhythmic contraction of the hypertrophic masseter can be observed on the right side
